# Editorial: Effects of microplastics on soil ecosystems

**DOI:** 10.3389/fmicb.2025.1686836

**Published:** 2025-09-22

**Authors:** Junqiang Zheng, Periasamy Dhevagi, Sesan Abiodun Aransiola, Shahid Iqbal, Yuzhe Wang

**Affiliations:** ^1^School of Life Sciences, Henan University, Kaifeng, China; ^2^Department of Environmental Sciences, Tamil Nadu Agricultural University, Coimbatore, India; ^3^Department of Microbiology, Faculty of Science, University of Abuja, Abuja, Nigeria; ^4^Kunming Institute of Botany, Chinese Academy of Sciences (CAS), Kunming, China; ^5^College of Juncao Science and Ecology, Fujian Agriculture and Forestry University, Fuzhou, China

**Keywords:** microplastics (MPLS), soil ecosystem, carbon cycling and sequestration, nitrogen transformation, microbial communities

Any plastics that are < 5 millimeters in size are called microplastics. They are miniature plastic particles and are increasingly recognized as a global environmental contaminant. They can originate from various sources, such as synthetic fibers from clothing, microbeads in personal care products and the breakdown of larger plastic debris. Microplastics in soil pose significant environmental risks, affecting ecosystems, plant growth, soil microbial communities and potentially human health. Understanding the effects of microplastics on soil microbial communities is crucial because these microbial communities play a vital role in maintaining soil fertility, supporting plant growth, and regulating greenhouse gas emissions. The accumulation of microplastics may physically alter soil structure, potentially affecting water retention, aeration, and the overall habitat for soil microbes. Microplastics could change the composition of soil microbial communities by preferentially adsorbing certain microbes over others, leading to shifts in the balance of bacteria, fungi, and other microorganisms as revealed in all the articles in this Research Topic.

There is increasing concern that microplastics may undergo degradation by microbial activity and subsequently accumulate within the soil food web, potentially leading to unknown consequences for soil ecosystem functions. The interaction between microplastics and agriculture is complicated and nuanced. Microplastics have the potential to disrupt symbiotic interactions between microbes and plants, such as mycorrhizal associations, which are essential for nutrient uptake and overall plant health. Moreover, microplastics can act as substrates for microbial colonization, possibly promoting the aggregation of microbial communities and facilitating the horizontal transfer of genetic material, including antibiotic resistance genes. Furthermore, the possibility of microplastics entering the food chain raises worries regarding food safety and human health. The broader implications of microplastic contamination for soil health and functionality remain complex and not yet fully understood, underscoring the need for further research to evaluate their long-term impacts on terrestrial ecosystems.

Plastic film mulching is widely used in cool, semi-arid agricultural regions, but the microbial mechanisms behind its effects across the entire growth cycle are not well understood. Kong et al. conducted a relevant and well-designed study that improves our understanding of how soil microbes respond to mulching at different growth stages of maize. Although the study relies on Biolog plates without sequencing data, its time-based design and integration with crop performance make it a valuable contribution. By linking microbial activity during the growing season—rather than single-time measurements—to crop yield, the research fills an important knowledge gap. To strengthen the paper, the authors could improve the statistical model, include more soil-related factors, and briefly address plastic residue concerns. The study shows that while mulching improves yield and water-use efficiency, microbial carbon metabolism may decline mid-season. To address this, the authors suggest adding organic carbon—a practical solution with immediate agricultural value. Overall, the research highlights that the relationship between mulching and soil microbes is complex and changes over time, cautioning against relying on simple, one-time assessments.

In a strong mechanistic study, Lai et al. advance the discussion on biodegradable microplastics. By including time-based and plant-focused measures, the study could reach “high-impact” status. The authors compare conventional (polyethylene, PE) and biodegradable (polylactic acid, PLA) microplastics and also examine aging—a key factor missing in current risk assessments of eco-friendly plastics. Their focus on rice, a globally important crop, connects general soil findings to food security systems. The study also links changes in dissolved organic carbon (DOC) to enzyme activity and microbial stability, showing how microplastics affect underground ecosystems. It challenges the idea that biodegradable microplastics are inherently safe. Instead, it shows that PLA fragments can briefly disrupt rice root systems, nutrient cycles, and greenhouse gas levels—risks that must be included in global standards for “agro-biodegradable” plastics. The findings shift the goal from “degrade quickly” to “degrade smartly”—plastics that release carbon in sync with microbes, neutralize acid by-products, and avoid nanofragment risks before use.

A study by Yi et al. reports a highly efficient di-n-butyl phthalate (DBP)-degrading bacterial strain, *Priestia megaterium* P-7, isolated from cotton fields in Xinjiang with long-term plastic-film mulching. The strain completely degraded 100 mg L^−1^ DBP within 20 h and tolerated up to 500 mg L^−1^ without a lag phase. Genomic analysis revealed a 5.57 Mb genome containing 28 genes involved in the sequential breakdown of DBP into mono-butyl phthalate, then phthalic acid, followed by transformation into protocatechuate, catechol, and finally integration into the TCA cycle via ring cleavage. Metabolomic data confirmed parallel β-oxidation and decarboxylation pathways. Optimization using Fe^2+^ (0.12 g L^−1^), sucrose (0.19 g L^−1^), and beef extract (9.92 g L^−1^) improved degradation efficiency to 78.5%. Strain P-7 also degraded other PAEs such as DMP, DEP, BBP, and DEHP, with higher efficiency for short-chain phthalates. It remained active across pH levels 5–9 and under high salinity. Its fast degradation rate, broad substrate range, and environmental resilience make it a promising candidate for bioremediation of PAE-contaminated agricultural soils.

In a split-plot field experiment, Xie et al. compared the effects of long-term polyethylene (PE) mulch fragments (~0.1% w/w) and one-season PBAT+PLA biodegradable mulch on rapeseed and soil microbiota in Chengdu Alfisols. PE did not affect seed yield or biomass but reduced soil nitrogen levels, suppressed Actinobacteria, and altered gene expression related to hydrocarbon and cellulose degradation while promoting ureolysis. PBAT-PLA increased rapeseed yield and biomass by improving soil moisture and nutrient availability (${\text{NH4}}_{4}^{+}$, ${\text{NO3}}_{3}^{-}$, DOC, DON). It also enhanced bacterial diversity, enriched beneficial genera (*Candidatus Udaeobacter, Acidibacter, Flavisolibacter*), and boosted genes involved in chitin and plastic degradation. Both mulch residues reduced microbial network complexity and stability, with PE causing more severe disruption. Structural equation modeling showed that PBAT-PLA created a strong positive soil–microbe–plant feedback (*r* = 0.91), while PE had neutral effects on plants but negative impacts on soil nitrogen. The study concludes that biodegradable mulch offers short-term agronomic benefits with less ecological disruption than PE. However, both types compromise microbial stability, highlighting the need for long-term monitoring and residue management. This is the first realistic field comparison between legacy PE and fresh biodegradable mulch in rapeseed cultivation, China's major oil crop. It challenges the assumption that biodegradable residues are harmless: while they improve yield and fertility, both mulch types weaken microbial network stability, with PE being significantly more damaging. The findings call for long-term studies with all types of plastic residues and dose optimization to balance productivity with ecosystem resilience.

In summary, film-mulched soils contain an effective *Priestia megaterium* P-7 strain that rapidly degrades DBP. Biodegradable PBAT-PLA films outperform conventional PE films by improving rapeseed yield, soil moisture retention, and nitrogen cycling, though both film types may reduce microbial network stability. Life cycle and economic assessments show that combining straw return with biodegradable mulching lowers global warming potential, acidification, and garlic production costs. Future research should focus on: (1) long-term field trials (>5 years) to track micro- and nanoplastic formation and assess ecological resilience; (2) multi-crop and multi-climate studies to evaluate the balance between yield gains and soil health; (3) chemo-omics analyses linking degradation by-products to microbial community changes; and (4) economic life cycle assessments that include on-farm biodegradation enhancers and residue recycling strategies.

